# Acute Hypoxemic Respiratory Failure in Immunocompromised Patients: Taking Aggressive Measures to Identify Etiology

**DOI:** 10.3389/fmed.2020.00433

**Published:** 2020-09-10

**Authors:** Jing Xu, Yuetian Yu, Jialin Liu

**Affiliations:** ^1^Department of Critical Care Medicine, School of Medicine, Rui Jin Hospital, Shanghai Jiao Tong University, Shanghai, China; ^2^Department of Critical Care Medicine, School of Medicine, Ren Ji Hospital, Shanghai Jiao Tong University, Shanghai, China

**Keywords:** non-invasive mechanical ventilation (NIV), hypoxeamia, highflow, respiratory failure, immunocompromised host

Immunocompromised patients often experience pulmonary complications especially acute respiratory failure (ARF) (1–3), the main cause of which is infection (4). To avoid increasing the risk of infection, non-invasive mechanical ventilation (NIV) is recommended as a first line treatment for immunocompromised patients (5, 6). However, recent studies have cast some doubts on the protective effects of NIV as a first-line treatment in immunocompromised patients with ARF (7–9). Meanwhile, a high-flow nasal cannula (HFNC) may potentially be a better alternative to NIV or standard oxygenation therapy (SOT) in immunocompromised patients due to its unique functions (4), but the results of studies of the effect of HFNC are also obscure (10–12). It has been determined that the identification of ARF etiology in immunocompromised patients currently contributes extensively to the survival rates of these patients.

Recently, Azoulay et al. (13) conducted a large, multinational prospective cohort study named the Efraim study, in which 1,611 critically ill immunocompromised patients with ARF were enrolled. They compared the intubation rate between four groups: standard oxygen only, HFNC only, NIV only, and both HFNC + NIV. Meanwhile, they also included patients who received first-line invasive mechanical ventilation (IMV) and all-cause hospital mortality rates were compared between these groups. The study illustrated that the use of NIV did not impact outcomes. Although HFNC reduced the intubation rate, it did not influence all-cause hospital mortality. However, more importantly, they found that the etiology of ARF had an impact on hospital mortality, acting as a vital confounder in the association between oxygenation strategy and mortality, and the study proved that patients with undetermined ARF have a very high mortality rate.

## What Causes the Negative Results of NIV and HFNC Despite their Obvious Advantages in Reducing Infection Compared to IMV?

The successful application of NIV or HFNC depends greatly on the selection of target patients and the optimal timing of the initiation of the study, as well as discontinuation of the first trial of NIV or HFNC. In the IVNIctus randomized controlled trial, immunocompromised patients received NIV treatment early, before they met IMV criteria. The authors were surprised to observe significant decreases in intubation rates and mortality rates in these patients compared with those determined in former studies (14). The delayed use of NIV was a pivotal predictor of NIV failure (15) and the mortality rate of patients increased dramatically after the failure of NIV (12, 13, 16). These results signal that it may be useful to move the timing and initiation of NIV ahead. Consistent with previous studies, the Efraim study demonstrated that the effect of intubation on mortality was determined by the timing of intubation and initial oxygenation strategy. After the failure of NIV, SOT, or HFNC, the odds of mortality increased compared to first-line IMV. To note, among these groups, patients with HFNC had higher risks of mortality after the failure of HFNC (13), which calls for the close supervision of patients with HFNC and timely conversion to IMV.

In addition, choosing oxygen strategies according to disease severity also exerts a great influence on endpoints. A recent systematic review and meta-analysis showed that NIV is a promising strategy in less severe patients (reflected by Simplified Acute Physiology Score II <60) (17). However, for more severe patients, NIV fails to show clear advantages over IMV (4). Similarly, as demonstrated in a recent study, the Sequential Organ Failure Assessment score is positively associated with HFNC failure, and there is a high possibility of HFNC failure in patients with ARF who initially had organ dysfunction (18). However, well-designed randomized controlled trials are still required, to further establish the predictors of HFNC failure in immunocompromised patients with ARF.

## Have We Done Our Best to Identify the Causes of ARF?

The increased mortality of patients with ARF of undetermined causes, as stated previously, may be ascribed to delays arising from a failure to recognize and treat a pulmonary complication. The study compared the diagnostic workup between patients with established causes of ARF and those with undetermined ARF etiology. The number of uses of fiberoptic bronchoscopy and bronchoalveolar lavage, as well as the type and number of uses of non-invasive diagnostic tests, were not significantly different between the two groups. Consequently, the study suggested that clinicians should make more effort to identify the etiology of ARF.

To avoid intubation, clinicians are reluctant to use IMV as the initial oxygenation strategy, which precludes the application of some useful diagnostic techniques. In the past, these methods may have gained limited benefits due to their poor diagnostic efficacy. However, emerging new technologies bring the potential of a more rapid and detailed resolution of etiology. Previously, the microbiological diagnosis was very challenging, due to the negative blood cultures created by the numerous opportunistic pathogens to which immunocompromised patients are susceptible (19). Nevertheless, with new diagnostic methods like nucleic acid sequencing (next-generation sequencing, NGS) and mass-spectrometry-based methods, the specificity and sensibility of pathogen detection has significantly increased (20). NGS was recently reported to show superior abilities in detecting *Mycobacterium tuberculosis*, anaerobes, and potentially Nocardia, and is less likely to be affected by prior antibiotic usage (20). Furthermore, 61% of NGS-positive cases in which the conventional method was inconclusive led to diagnosis modification (20). In a multicenter, prospective study, 101 immunocompromised adults were enrolled and underwent both standard procedures (including blood culture, serological diagnosis, antigen detection, PCR, and direct examination of specimens) and next-generation sequencing (NGS) for the first line diagnosis of infection. The results detected a significantly higher proportion of patients with infection by NGS than that by standard procedures (SP) (36 vs. 11%). The viruses and bacteria that were only identified by NGS, but not SP, mainly included rhinovirus A, adenovirus B *Bacteroides fragilis, Streptococcus pneumoniae, Acinetobacter baumannii*, and *Pseudomonas* sp, most of which are opportunistic pathogens (19). More recently, mass-spectrometry-based methods have revolutionized pathogen identification (21). With the advantages of extreme rapidity (a matter of minutes), cost efficiency, and high specificity, the matrix-assisted laser desorption/ionization time of flight mass spectrometry has been demonstrated to outperform conventional methods in identifying anaerobic bacteria, mycobacteria, and fungi, and occasionally, in predicting the antibiotic phenotypic resistance profile (22–24). With these methods, patients may benefit from more appropriate therapy. Grumaz et al. found that NGS diagnostics would have led to a change in antimicrobial therapy in 53% of patients based on retrospective assessment by experts. The patients with adequate treatment had higher survival rates than patients who were inadequately treated, indicating that NGS may improve the survival of sepsis by assisting physicians in establishing appropriate antibiotic treatment regimens (25).

In addition to microbiological diagnosis, other new techniques are increasingly used in the identification of etiology. For instance, endobronchial ultrasound-guided transbronchial needle aspiration (EBUS-TBNA) was recently shown to be a useful procedure for the diagnosis of sarcoidosis. Compared to conventional TBNA, EBUS-TBNA has a markedly higher diagnostic yield in sarcoidosis (74.5 vs. 48.4%) (26).

With these advanced diagnostic techniques, clinicians are more likely to identify the real cause of ARF and tailor specific treatment protocols for different patients. In this case, clinicians are expected to weigh the pros and cons of IMV more carefully, from a new perspective. As a matter of fact, with improvement in intensive care unit management, intubation is not as fatal as we had previously thought for immunocompromised patients. A study conducted by Lemiale et al. found that the mortality of intubated patients has decreased notably, whereas other determinants of mortality including the ability to identify ARF etiology are still heavily impactful (10). Taking precautions to minimize infection risks in patients with immunosuppression is truly essential, but it may be time to be confident in controlling infection, and give priority to identifying ARF etiology when choosing an oxygenation strategy, possibly including intubation for diagnostic procedures. At the same time, an aggressive diagnostic strategy should be considered if necessary. Nonetheless, we still need more randomized controlled trials to provide thorough evidence to guide us in this issue.

## Conclusions

In conclusion, HFNC is a potential oxygenation therapy to be used in immunocompromised patients but more evidence is needed to determine the target patients as well as the optimal timing of initiation and discontinuation of HFNC. More importantly, clinicians should give priority to identifying the etiology of ARF by implementing aggressive diagnostic methods instead of refusing intubation merely to reduce infection risks.

## Author Contributions

JX: conception, design, provision of study materials or patients, and data analysis and interpretation. YY and JL: administrative support. All authors: manuscript writing, collection and assembly of data, and final approval of manuscript.

## Conflict of Interest

The authors declare that the research was conducted in the absence of any commercial or financial relationships that could be construed as a potential conflict of interest.

## References

[1] Gristina GR Antonelli M Conti G Ciarlone A Rogante S Rossi C. Noninvasive versus invasive ventilation for acute respiratory failure in patients with hematologic malignancies: a 5-year multicenter observational survey. Crit Care Med. (2011) 39:2232–9. 10.1097/CCM.0b013e3182227a27 21666446

[2] Azoulay E Lemiale V Mokart D Pene F Kouatchet A Perez P Acute respiratory distress syndrome in patients with malignancies. Intensive Care Med. (2014) 40:1106–14. 10.1007/s00134-014-3354-0 24898895

[3] Canet E Osman D Lambert J Guitton C Heng AE Argaud L. Acute respiratory failure in kidney transplant recipients: a multicenter study. Crit Care. (2011) 15:R91. 10.1186/cc10091 21385434 PMC3219351

[4] Wang T Zhang L Luo K He J Ma Y Li Z. Noninvasive versus invasive mechanical ventilation for immunocompromised patients with acute respiratory failure: a systematic review and meta-analysis. BMC Pulm Med. (2016) 16:129. 10.1186/s12890-016-0289-y 27567894 PMC5002326

[5] Schonhofer B Kuhlen R Neumann P Westhoff M Berndt C Sitter H. Clinical practice guideline: non-invasive mechanical ventilation as treatment of acute respiratory failure. Dtsch Arztebl Int. (2008) 105:424–33. 10.3238/arztebl.2008.0424 19626185 PMC2696903

[6] Keenan SP Sinuff T Burns KE Muscedere J Kutsogiannis J Mehta S. Clinical practice guidelines for the use of noninvasive positive-pressure ventilation and noninvasive continuous positive airway pressure in the acute care setting. CMAJ. (2011) 183:E195–214. 10.1503/cmaj.100071 21324867 PMC3042478

[7] Depuydt PO Benoit DD Roosens CD Offner FC Noens LA Decruyenaere JM. The impact of the initial ventilatory strategy on survival in hematological patients with acute hypoxemic respiratory failure. J Crit Care. (2010) 25:30–6. 10.1016/j.jcrc.2009.02.016 19682849

[8] Frat JP Ragot S Girault C Perbet S Prat G Boulain T. Effect of non-invasive oxygenation strategies in immunocompromised patients with severe acute respiratory failure: a post-hoc analysis of a randomised trial. Lancet Respir Med. (2016) 4:646–52. 10.1016/S2213-2600(16)30093-5 27245914

[9] Wermke M Schiemanck S Hoffken G Ehninger G Bornhauser M Illmer T. Respiratory failure in patients undergoing allogeneic hematopoietic SCT–a randomized trial on early non-invasive ventilation based on standard care hematology wards. Bone Marrow Transplant. (2012) 47:574–80. 10.1038/bmt.2011.160 21927036

[10] Lemiale V Resche-Rigon M Mokart D Pene F Argaud L Mayaux J. High-flow nasal cannula oxygenation in immunocompromised patients with acute hypoxemic respiratory failure: a groupe de recherche respiratoire en reanimation onco-hematologique study. Crit Care Med. (2017) 45:e274–80. 10.1097/CCM.0000000000002085 27655324

[11] Coudroy R Jamet A Petua P Robert R Frat JP Thille AW. High-flow nasal cannula oxygen therapy versus noninvasive ventilation in immunocompromised patients with acute respiratory failure: an observational cohort study. Ann Intensive Care. (2016) 6:45. 10.1186/s13613-016-0151-7 27207177 PMC4875575

[12] Amado-Rodriguez L Bernal T Lopez-Alonso I Blazquez-Prieto J Garcia-Prieto E Albaiceta GM. Impact of initial ventilatory strategy in hematological patients with acute respiratory failure: a systematic review and meta-analysis. Crit Care Med. (2016) 44:1406–13. 10.1097/CCM.0000000000001613 26909503

[13] Azoulay E Pickkers P Soares M Perner A Rello J Bauer PR. Acute hypoxemic respiratory failure in immunocompromised patients: the Efraim multinational prospective cohort study. Intensive Care Med. (2017) 43:1808–19. 10.1007/s00134-017-4947-1 28948369

[14] Lemiale V Mokart D Resche-Rigon M Pene F Mayaux J Faucher E. Effect of noninvasive ventilation vs oxygen therapy on mortality among immunocompromised patients with acute respiratory failure: a randomized clinical trial. JAMA. (2015) 314:1711–9. 10.1001/jama.2015.12402 26444879

[15] Benoit DD Depuydt PO. Non-invasive ventilation in patients with hematological malignancies: the saga continues, but where is the finale? Intensive Care Med. (2010) 36:1633–5. 10.1007/s00134-010-1949-7 20607515

[16] Huang HB Peng JM Weng L Liu GY Du B. High-flow oxygen therapy in immunocompromised patients with acute respiratory failure: a review and meta-analysis. J Crit Care. (2018) 43:300–5. 10.1016/j.jcrc.2017.09.176 28968525

[17] Wang T Liu G He K Lu X Liang X Wang M. The efficacy of initial ventilation strategy for adult immunocompromised patients with severe acute hypoxemic respiratory failure: study protocol for a multicentre randomized controlled trial (VENIM). BMC Pulm Med. (2017) 17:127. 10.1186/s12890-017-0467-6 28931394 PMC5607592

[18] Kim WY Sung H Hong SB Lim CM Koh Y Huh JW. Predictors of high flow nasal cannula failure in immunocompromised patients with acute respiratory failure due to non-HIV pneumocystis pneumonia. J Thorac Dis. (2017) 9:3013–22. 10.21037/jtd.2017.08.09 29221274 PMC5708421

[19] Parize P Muth E Richaud C Gratigny M Pilmis B Lamamy A. Untargeted next-generation sequencing-based first-line diagnosis of infection in immunocompromised adults: a multicentre, blinded, prospective study. Clin Microbiol Infect. (2017) 23:574 e571–4. e576. 10.1016/j.cmi.2017.02.006 28192237

[20] Miao Q Ma Y Wang Q Pan J Zhang Y Jin W. Microbiological diagnostic performance of metagenomic next-generation sequencing when applied to clinical practice. Clin Infect Dis. (2018) 67:S231–40. 10.1093/cid/ciy693 30423048

[21] Goh C Knight JC. Enhanced understanding of the host–pathogen interaction in sepsis: new opportunities for omic approaches. Lancet Respir Med. (2017) 5:212–23. 10.1016/S2213-2600(17)30045-0 28266329

[22] Idelevich EA Reischl U Becker K. New microbiological techniques in the diagnosis of bloodstream infections. Dtsch Arztebl Int. (2018) 115:822–32. 10.3238/arztebl.2018.0822 30678752 PMC6369238

[23] Luethy PM Johnson JK. The use of matrix-assisted laser desorption/ionization time-of-flight mass spectrometry (MALDI-TOF MS) for the identification of pathogens causing sepsis. J Appl Lab Med. (2019) 3:675–85. 10.1373/jalm.2018.027318 31639735

[24] Tran A Alby K Kerr A Jones M Gilligan PH. Cost savings realized by implementation of routine microbiological identification by matrix-assisted laser desorption ionization-time of flight mass spectrometry. J Clin Microbiol. (2015) 53:2473–9. 10.1128/JCM.00833-15 25994167 PMC4508454

[25] Grumaz S Vainshtein Y Stevens P Glanz K Decker SO Hofer S. Enhanced performance of next-generation sequencing diagnostics compared with standard of care microbiological diagnostics in patients suffering from septic shock. Criti Care Med. (2019) 47:e394–402. 10.1097/CCM.0000000000003658 30720537 PMC6485303

[26] Gupta D Dadhwal DS Agarwal R Gupta N Bal A Aggarwal AN. Endobronchial ultrasound-guided transbronchial needle aspiration vs conventional transbronchial needle aspiration in the diagnosis of sarcoidosis. Chest. (2014) 146:547–56. 10.1378/chest.13-2339 24481031

